# Performance Evaluation of Direct-Link Backhaul for UAV-Aided Emergency Networks

**DOI:** 10.3390/s19153342

**Published:** 2019-07-30

**Authors:** German Castellanos, Margot Deruyck, Luc Martens, Wout Joseph

**Affiliations:** 1 Department of Electronics Engineering, Colombian School of Engineering, Bogota 111166, Colombia; 2 Department of Information Technology, IMEC-Ghent University, 9052 Ghent, Belgium

**Keywords:** UABS, backhaul, UAV, disaster scenarios, millimeter wave

## Abstract

Today’s wireless networks provide us reliable connectivity. However, if a disaster occurs, the whole network could be out of service and people cannot communicate. Using a fast deployable temporally network by mounting small cell base stations on unmanned aerial vehicles (UAVs) could solve the problem. Yet, this raises several challenges. We propose a capacity-deployment tool to design the backhaul network for UAV-aided networks and to evaluate the performance of the backhaul network in a realistic scenario in the city center of Ghent, Belgium. This tool assigns simultaneously resources to the ground users—access network—and to the backhaul network, taking into consideration backhaul capacity and power restrictions. We compare three types of backhaul scenarios using a 3.5 GHz link, 3.5 GHz with carrier aggregation (CA) and the 60 GHz band, considering three different types of drones. The results showed that an optimal UAV flight height (80 m) could satisfy both access and backhaul networks; however, full coverage was difficult to achieve. Finally, we discuss the influence of the flight height and the number of requesting users concerning the network performance and propose an optimal configuration and new mechanisms to improve the network capacity, based on realistic restrictions.

## 1. Introduction

In normal circumstances, the cellular network is quite reliable and provides connectivity for users in cities and rural areas with high quality and speed. However, they are not exempt from failing during emergencies such as storms, earthquakes, tornados, bushfires, tsunamis or even terrorist attacks. In these cases, people tend to communicate more with their relatives to let them know if they are safe; as a result, cellular networks tend to saturate. Besides, the emergency itself could damage the system to the point that there is no land-based network to communicate. This was the case of the magnitude 7.0 2010 Haiti’s earthquake. For two days, the cellular network of the two mobile providers was offline. By the third day, only 70% of the cellular network could be re-established [1,2]. Later in 2017, Hurricane Maria in Puerto Rico took down 95% of the land-based network on September 21. One month later, 67.4% of the network was still out of service, and by the end of the year, even 36.0% of the grid was down according to [3]. Likewise, in the Sulawesi earthquake and tsunami in Indonesia on September 2018, 1678 base stations were damaged equally to 40.02% of the land-based network [4]. These are examples of emergencies that destroyed the land-based system severely. A promising solution that is broadly investigated [5,6,7,8,9,10] is the usage of unmanned aerial vehicles (UAVs) to aid the land-based network that suffered from congestions or physical failure. A base station (BS) is mounted on a UAV, which is positioned close to or above the affected area and provides mobile communications to the uncovered users. These UAVs are called unmanned aerial base stations (UABSs). Though this is a promising solution, it has several practical challenges. These challenges could be divided into two groups related to (i) the access network where the UABSs provide a connection to the users and (ii) the backhaul link between the UABSs and the core network (CN). Although several studies have addressed the access network [10,11,12,13,14,15] as will be discussed in Section 2, limited work has been done on the backhauling.

This paper proposes a backhauling architecture for a UABS network. Its performance is evaluated in a scenario of a real 3D model of Ghent city in Belgium. To this end, the capacity-based deployment tool described in [5] has been extended with the proposed backhaul architecture. To the best of the author’s knowledge, a deployment tool for UAV-aided networks that accounts for backhaul connectivity in a realistic scenario does not exist. The novelty of this proposal is that the allocation of ground users is done simultaneously to the allocation of the backhaul resources, accounting for both capacity and power constraints.

The remainder of this paper is organized as follows. In Section 2, a review of the state-of-art of UABS in emergency networks is presented for both the access and backhaul network. Section 3 outlines the role of the backhaul in these networks and proposes three scenarios that will be studied based on the methodology described in Section 4. Section 5 discusses the results of the simulation for a realistic scenario in the city of Ghent, Belgium. Finally, in Section 6, the paper is concluded and future work is presented.

## 2. UAV Serving Ground Users

An underlying network architecture for UAVs is presented in [15]. It describes and defines two categories of links: The control and non-payload (CNPL) and the data links. The first one should provide a full-duplex, low latency, high reliability and secure connections; usually with low data rates, for the usage of the UAV. Whereas the latter one is highly dependent on the role of the UAV. In this architecture, neither access nor backhaul links are described. When the application is using UAVs to aid wireless networks, they are called UABS. In this case, these links are divided into three different types: Access, relay and backhaul. 

The problem of using UAVs to support ground communications in disaster scenarios has been covered by diverse authors; however, their relation and constraints with the backhaul are not widely explored. Mazaffari et al. present in [16] one of the most extensive reviews of UAV in wireless networks. It covers various potential application including disaster scenarios, 5G BSs, mmWave communications, relaying and Internet of Things (IoT) applications. [17] proposes a mathematical framework for 3D cellular network based on UABS. Instead, Gupta et al. collected in [18] a detailed survey into the network-side challenges including physical, data link and network layers from the perspective of UAV networks. Cicek et al. in [10] present a taxonomy classification of location optimization solutions, which includes a study of 124 papers related to UABS. Its taxonomy comprises parameters of how the location of the UABS is done statically or dynamically, number of UABS, among others. The backhaul constraints are considered only in static scenarios with exact or PSH solutions. In [19], Zeng et al. describe the potential and challenges of using UAV as users of the cellular networks and includes using it to support backhaul network in case of a disaster. Different approaches are discussed from a 2D joint trajectory and scheduling optimization method for only one drone up to multiple relaying users equipment (UEs) to extend the coverage area. However, the backhaul is considered only between the core network and a disconnected BS by the usage of multiple UAVs that do not serve users [7]. Reference [20] presents a method to deploy and optimize UABS locations based in a distributed optimization model, to maximize coverage quality, serving time and minimize interference influence. Wu et al. present a 2D trajectory design using a tradeoff model between delay, power and throughput, concluding that the average throughput increased as the speed of the UAV increases at expenses of the delay [21]. Reference [22] introduces a 3D location mixed-integer non-linear programming algorithm for only one UABS being served by a terrestrial macro-cell network as a backhaul using 2 GHz frequency. Mozaffari et al. [14] present a study of multiple UABSs 3D placements for serving users maximizing the coverage area; however, it does not include backhaul limitations. In [9], Deruyck et al. propose a deployment tool to investigate network performance during a disaster, but it does not account for the backhaul. Similar, [8] presents a public safety heterogeneous network with two tiers, the microcell and the UAV network, and their backhaul links are considered to be infinite. Kawamoto et al. in [13] present the first known trial of a UAV serving users using WiFi resource management. They evaluate the impact of motion, antenna directivity and resource allocation in the experiment. Lime Microsystems demonstrate a 3G call for a drone-mounted lightweight base station with a weight less than 2 kg [23]. In [12], the results of a UAV relaying a user using an existing long term evolution (LTE) network are presented. The increase in user throughput when the flying relay is in operation is shown. [24] presents the challenges of using millimeter-wave communications to connect UAVs either for access or for backhaul and focus on the challenges of detection, positioning, interference mitigation and spectrum sharing in UAVs. [25] proposes a stochastically geometry model to perform backhaul scenario for UABS in 2.6 and 73 GHz. In [26] a study of the backhaul at 3.5 and 30 GHz is done. 

## 3. The Architecture of Backhaul for UABS in Emergency Networks

### 3.1. Backhaul Architecture

**Figure 1:**
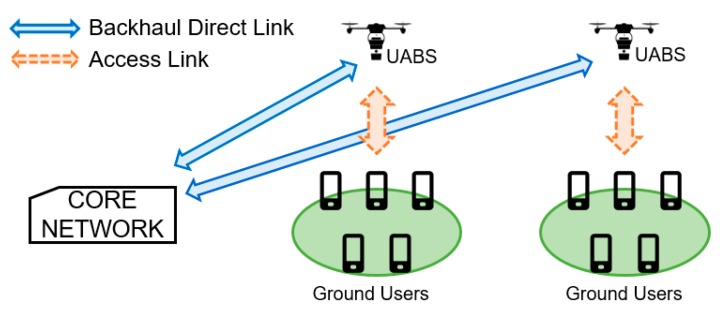
Direct backhaul scenario for an emergency network using unmanned aerial base stations (UABSs).

We proposed a general architecture for UAV-aided networks as illustrated in Figure 1. It is based on the architectures described in [7,10,12,15,21]. The access network, indicated in orange, uses the 2.6 GHz frequency and connects the users to the UABSs through 3G LTE femtocell base station technology. This provides essential communication like voice, text and limited data in case of a disaster. A detailed explanation of the access network in this architecture could be found in [5]. Understanding the capacity requirements of the access network, as well as the restrictions in emergencies, is vital to determine the appropriate backhaul network. We propose a backhaul link configuration called direct link. In this backhaul configuration, the UABSs have a direct wireless connection with the CN, as shown by the blue arrow. This configuration is similar for the BSs of the terrestrial network, where microwave links are typically used [27] and are therefore considered as out-of-band backhaul links having the advantage of minimal or none interference with the access network. The direct link uses long term evolution (LTE) release 11, which can manage carrier aggregation. The usage of a known technology like this, aids in the management of resource allocation parameters used in this architecture, defining the UAV like a flying user in an LTE network. We proposed to use two different frequency bands namely the 3.5 GHz and 60 GHz bands that will be described in the next subsection. The radio frequency parameters are also described in Section 4.2.

Our architecture used LTE-advanced time division duplexing (TDD) in the backhaul network. This configuration aids in the usage of TDD time synchronization that will minimize intra interference in the proposed architecture [28]. Moreover, because our study assumes that all the land-based cellular network is offline; further inter-cell interference is ruled out too. Hence, no interference is considered in our architecture.

### 3.2. Frequency Selection

Backhaul frequency usage serving international mobile telephony (IMT) is divided into out-of-band or in-band, having different frequencies or sharing the same frequency band respectively. The first one is the most commonly used due to the versatility of the network and the independence between the access and the backhaul network, which leads to minimal interference [27]. On the other hand, the in-band perspective is included in LTE Release 10 as a relay mechanism to support evolved NodeB (eNBs) that have suffered from lost backhaul connectivity and should rely on a Donor eNB (DeNB) to have access to the CN [29]. In-band backhauling is an attractive option due to the fast reconfiguration time and low operational cost but imposes interference management and resource allocation challenges. Siddique et al. present a comparison of the different frequencies used in the backhaul and the benefits of using sub-6 GHz frequencies for UABS arrangements [27]. The authors conclude that licensed sub-6 GHz frequencies are the best compromise between coverage and capacity, allowing easy operation and management even in non-line-of-sight (NLoS) conditions. However, limited and expensive spectrum and the need for interference management are considered as significant drawbacks for traditional IMT sub-6 GHz bands.

To overcome the issue of the limited spectrum, we proposed the use of the 3.5 GHz band, which is still not broadly occupied, as it is the case for the 800, 900, 1800, 1900 and 2600 MHz bands. In the range of the 3400 to 3600 MHz the band 22 is available for frequency division duplexing (FDD) services, the 42 or 43 bands for TDD and the n78 band for 5G New Radio (5G_NR) [30,31]. The main reason for choosing this band is the ability to work with either an out-of-band or in-band configuration, as well as the low occupancy of this band, the licensed protection and the capacity-coverage trade-off [32]. Recent 5G trials have shown data rate peaks of 10.4 Gbps using 200 MHz of the 3.5 GHz band, proving to have the potential to fulfill backhauling requirements [33]. 

Millimeter-wave frequencies have also been studied for 5G radio access networks, due to the ability to support large bandwidth (several GHz) but with high path loss attenuations. There are mainly two groups for mmWave frequencies studied for IMT: The 50 GHz group (45.5–47; 47.0–47.2; 47.2–50.2; 50.4–52.6) and the 80 GHz Group (66–76; 71–76; 81–86). However, most of them are studied for the access network. Some research is being done for fixed wireless access (FWA) and backhaul in the mmWave V-band. The 60 GHz band (57–66 GHz) is a promising candidate for backhauling, due to unlicensed or light-licensed operations, no spectrum shortage, high capacity, interference resistance, frequency reuse ability thanks to its narrow beam directivity and the small size of antennas that can be easily mounted in the UAV [34]. Recent trials in Hungary using 60 GHz for fixed wireless broadband have demonstrated that V-Band could support up to 1 Gbps connectivity [35].

We proposed to use the 60 GHz band (57–66 GHz) band for the backhaul link because it supports 9 GHz of unlicensed bandwidth that could be used even with low spectrum efficiency to tolerate the high attenuation of this band and still provide the data rates needed in disaster scenarios. 

## 4. Methodology

In this section, the emergency network and scenario definitions for the simulation are presented. Then, the UABS path loss model and link budget parameters are defined, following by the description of the types of drones used for the backhaul analysis. Finally, the simulation algorithm for UAV-aided backhaul network planning was proposed. 

### 4.1. Emergency Network

**Figure 2:**
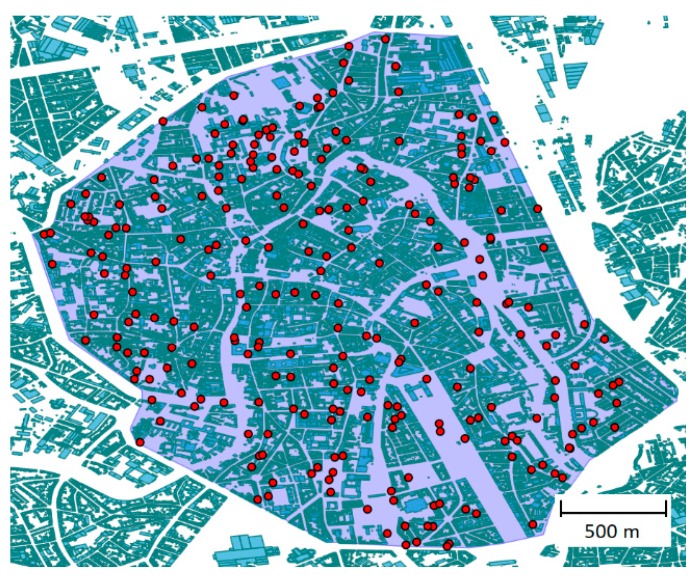
The city center of Ghent with 260 ground users in red circles.

Under normal conditions during the day, 260 concurrent users are possible at the maximum traffic at 5 pm in the city of Ghent, according to real data from a Belgian mobile operator [5]. For this study, we assumed a worst-case scenario where the entire land-based network was down in the city center of Ghent (6.85 km^2^), Belgium. This means that the UABSs will support all the communications and all the users’ traffic will go through this network architecture. Figure 2 shows the city center of Ghent with the 260 users uniformly distributed in red circles with indoor and outdoor locations having the same probability. Users inside buildings are positioned at half of the building height. 

The traffic distribution is essential to define the traffic model that each UABS has to serve in the access network and consequently, in the backhaul network. For these scenarios, only voice (64 kbps) and data (1 Mbps) traffic was used, and its distribution was based on information from the operator. New active users will be able to connect once an active user goes idle. Hence, 260 voice/data connections will have to remain active during the whole intervention, which is one hour. However, the connectivity of a user and the activation of a UABS will depend on the available capacity of the backhaul network. When the emergency occurs, a facility truck with the UABS goes to the center of the emergency area and deploys the UABS. They will fly to its defined location and then start to serve ground users. Finally, handover procedures and inter-cell interference in the access network are assumed too. In Table 1, the simulation configuration for the scenarios is presented.

### 4.2. Scenarios Definition

We proposed three different scenarios for serving backhaul to the drones. The first scenario (I) considers connecting all the UABS to the CN through LTE-advanced technology using a 3.5 GHz channel with a 20 MHz bandwidth. A first in first out (FIFO) based algorithm for resource block (RB) allocation is implemented where user resources are assigned simultaneously based on the backhaul constraints [36]. The RB is the tiniest component of resources that can be allocated to devices in LTE. Each RB has 12 subcarriers and seven symbols equivalent to a one time slot in the LTE frame. The amount of RB is dependent on the carrier bandwidth; for 20 MHz 100 RB are used [37]. We also assumed no interference between the backhaul network (UABS to CN) and the access network (users to UABS). For the second scenario (II), carrier aggregation (CA) was included, aggregating five carrier components (CC) to complement a total of 100 MHz equivalent to 500 RBs. The third scenario (III), was a direct link scenario using mmWave connectivity. In this scenario, UABSs were connected to the CN using a 60 GHz link. The radio and link budget parameters for the simulation are presented in Table 2. The 3.5 GHz link used LTE-advanced orthogonal frequency division multiple access (OFDMA) configuration.

### 4.3. Path Loss Models

The 3GPP TR 36.777 draft [38] outlines urban and rural environments for UAV implementations over LTE release 15 networks. The standard proposes system level parameters and channel models, which are included in our proposed scenario. For the 3.5 GHz band, we used the model in [38] based on 3GPP TR 38.901 [39], which accounts for 3D scenarios and is optimal for our simulation tool requirements. Shi et al. in [26] use this model in the 3.5 GHz band. Therefore, we proposed to use the urban macro (UMa) environment path loss model, which is described in Equations (1) and (2) for the line-of-sight (LoS) and NLoS respectively; where f_c_ is in GHz, d_3D_ and h_drone_ in meters. The shadow fading standard deviation σ_SF_ was 4 dB and 6 dB for LoS and NLoS, respectively. This model is valid for distances up to 4.5 km, and increases as the h_drone_ increases [26]. One of the advantages of using this model is its applicability to frequencies up to 100 GHz; hence, it will also be used for the 60 GHz band simulations. The path loss $PL_{UMa-LOS}$ in LoS and $PL_{UMa-NLOS}$ in NLoS conditions (in dB) is calculated as follows [34,35]:

(1) $$PL_{UMa-LOS}=28+22\log_{10}\left(d_{3D}\right)+20\log_{10}\left(f_{c}\right)$$

(2) $$PL_{UMa-NLOS}=13.54+39.08\log_{10}\left(d_{3D}\right)+20\log_{10}\left(f_{c}\right)-0.6\left(h_{drone}-1.5\right)$$

### 4.4. UAV Description

The types of drones used in the simulation are essential to determine the performance of the solution apart from the network parameters. We assumed that the access and backhaul network equipment weighs about 1.5 kg; hence, the selection of drones are based on this parameter [40]. We proposed three types of drones for these simulations. The first type of drone was a commercial off-the-shelf hexacopter drone like the DJI F550, which its size and battery capacity were small and its flight time was approximately 15 min [41]. The second drone was a professional quadcopter like the MD4-100 able to carry more than 1 kg of payload and with enough battery capacity to flight nearly 45 min with the described payload [42]. The third drone was a Harris H4 Hybrid drone, which combined a traditional quadcopter drone with a 48 V battery with an H2000 generator of 1.8 KWatts. Both will add together a total of 37.5 Ah per hour, and with a consumption of 1.5 L/h from a four-liter tank, the total capacity available will be 100 Ah [43]. From the facility, 1500 drones were available to serve the emergency; so when a drone was running out of battery, a new drone from the facility flight to the old drone’s position and replaced it until 1500 drones were used. Backhaul handover was considered seamless because it would happen less than four times during the intervention. All drone specifications are presented in Table 3.

### 4.5. Deployment Tool and Algorithms

The tool used in this paper was an extension of the one used in [5]. The novelty of this tool included modifications to the implementation of LTE radio resource allocation using RB to manage the radio resources and the capacity of the backhaul and the power consumption model for LTE femtocell BSs. The tool, implemented in Java, presents four different phases to achieve the full network simulation. 

**Figure 3:**
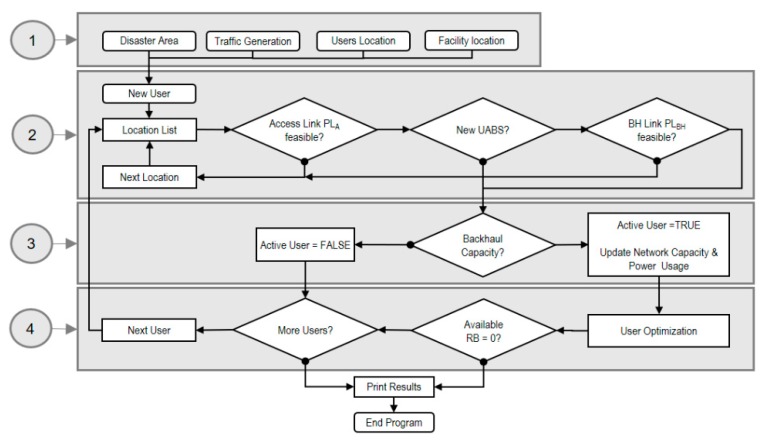
Flow diagram of the algorithm implemented for backhaul analysis.

First, the tool generates a realistic traffic scenario of the users with a uniform distribution in the studied area, assigns their locations and sets up a facility in the center of the considered area (Step 1 in Figure 3). Second, a list of possible UABS locations is assigned above of each user in the network at the configured flight altitude. Then, for each user, this list is organized based on the signal-to-noise-ratio (SNR) of the link between the user and the UABS locations. Then, for each link, it evaluates the access-link viability by calculating if the SNR is sufficient for the modulation and coding schemes (MCS) of the technology. For the UABS location with the best SNR, the tool asks if this UABS is a new one or an active one. If it is a new one, the algorithm evaluates the backhaul-link viability by checking the SNR and the MCS similarly to the access-link. If some of the links are not viable, it continues to the next UABS possible location and repeats the link evaluation process (Step 2 in Figure 3). Third, after evaluating the access and backhaul link viabilities, it proceeds to assess based on the capacity constraints. The algorithm calculates if the available backhaul resource blocks are enough for the new bit rate of the UABS. If the link capacity is feasible, the user is assigned to that UABS and finally, the used network capacity and power usage values are updated (Step 3 in Figure 3). Unlike [44], where there is a fixed number of UABS, our algorithm assigns UABS when they are needed, based on the restriction of the facility. Fourth, each new user is subject to the optimization of the active UABS to reduce transmission power, minimize flight time of the UABS and reduce the number of RB used. The algorithm finishes when all users are served or when the RBs are fully utilized (Step 4 in Figure 3). Finally, the tool collects the result variables and prints them for easy analysis.

To achieve stability in the simulation results, we ran the tool and obtained stability in 65 runs per scenario. The number of runs where stability was found depends on the deviation of the averaged values compared to the average values of five investigated values in 100 runs (number of locations, number of users, number of drones, mean flight time and mean capacity). We defined stability when the average value of the variables in specific runs had a deviation of ±0.5% of the average value in 100 runs. We looked for the number of runs for all values that fulfilled this criterion and we found it to be 65. 

## 5. Results

In this section, we introduced the simulation results based on the tool described above. Unlike in [5], that the analysis was focused on how many UABS were needed to support the disaster; here we focused the study on the behavior of the network when there were capacity restrictions in the backhaul and different frequencies to evaluate. For this, we divided the results in the three backhaul scenarios starting with the 3.5 GHz band with no CA, then with the 3.5 GHz with CA and finishing with the 60 GHz band. Besides, we collected the results of 21 variables in groups related to the UABS, the users, the network and backhaul capacity and power consumption.

### 5.1. Scenario I: LTE at 3.5 GHz No Carrier Aggregation

**Figure 4:**
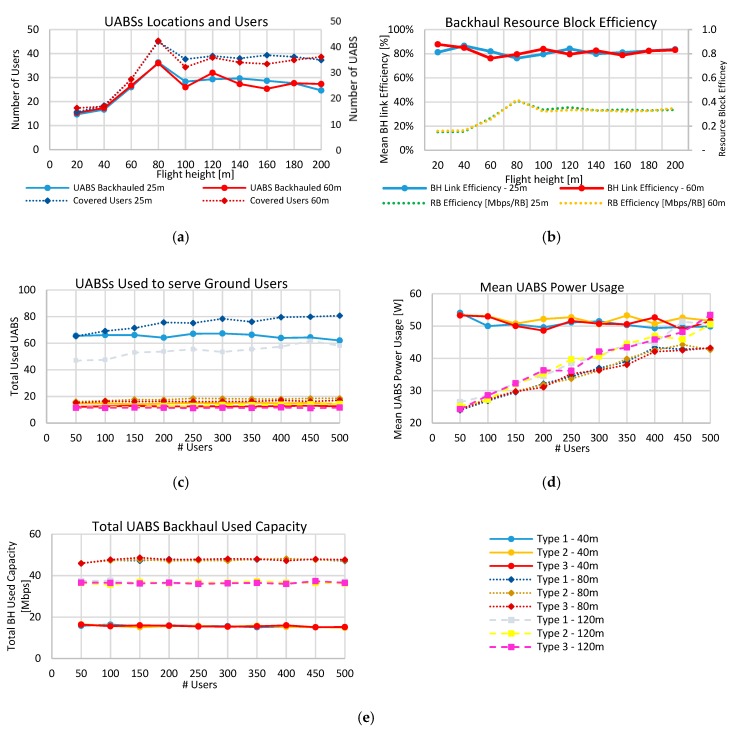
Evaluation results for the Scenario I. (**a**) Flight performance of UABSs. (**b**) Backhaul resource block performance. (**c**) UABS needed for different types of drones. (**d**) Power usage for different types of drones. (**e**) Backhaul network utilization for different kinds of drones.

We evaluated the performance of the network varying the flying height of the UABSs. The values assessed go from 20 m to 200 m above the ground level. We also studied the implication of the facility antenna height and compared an antenna of 25 m against a 60 m antenna using a type 1 drone. In Figure 4a, the required number of UABSs and the served users is presented. It can be seen that if the flight height was low, the number of backhauled UAVs was small due to the buildings that were present in the area. As the altitude increases, the number of line-of-sight (LoS) links increased and the viability increased leading to more suitable connections at 80 m serving around 45 users. This value was optimal and worked under the maximum limitations from the Belgian Civil Aviation Authority, which was 90 m for the considered type of drones [45]. Above this altitude, the connected number of UABSs decreased slightly due to the distance between the facility antenna and the UABS. It is vital to notice that the facility antenna height hardly affected the performance of the number of backhauled UABS only in values less than 60 m. Above that altitude, the UABS-users connectivity defined more the performance. Besides, it can be seen that usually, only 45 users were served. This value could not be increased because all of the 100 RBs were allocated. Figure 4b presents an assessment of the RB efficiency for the backhaul. If all the connection used the best MCS (64QAM-2/3), the maximum bandwidth achievable would be 72 Mbps. However, the actual bandwidth was 41.3 Mbps, equivalent to 0.4 Mbps per RB (dotted lines in Figure 4b), equal to an MCS of 16QAM-2/3.

Moreover, the maximum of 72 Mbps could hardly be achieved due to the resource block allocation, i.e., a user requesting 1 Mbps will need two RB of 720 kbps each, leading to 1.4 Mbps allocated but only 1 Mbps used (69.4% efficiency). This efficiency could be improved if more user’s requests are assigned in the available backhaul RB with divers MCS. Our results show that efficiency was nearly 81.9% ± 3.2%, as shown in the straight lines of Figure 4b. 

Next, we evaluated the performance of users (50–500 users) asking for resources in the network using the three types of drones (see Table 3) at three different altitudes (40, 80 and 120 m). Figure 4c,d,e present these results. In Figure 4c, the number of UABS needed depended on the type of drone used, where the worst case was with the Type 1 drone. Type 2 and hybrid type behaved similarly because the autonomy per drone was more than one hour, which was the considered intervention time. Hence similar results were obtained but with a slight increment from the Type 2 (16 hybrid drones to 18 Type 2 drones) mainly because of the speed of the drone. The average power used by UABSs as a function of the served users is presented in Figure 4d. The power consumption is the sum of the energy used by the flight from the facility to the serving location and return, plus the power for the transmission of the access and the backhaul network [46]. The averaged power consumption was almost independent of the type of drone used, and because the majority of the consumption was used in radio transmissions, it was proportional to the requesting users. At the optimal height of 80 m, a lower amount of power was consumed because the UABSs have to transmit less power to achieve the same bit rate due to better LoS links and lower flight times to the serving position. In Figure 4e, we evaluated the backhaul network performance. The results show that the network was stable independent of the number of users. As seen in the UABS altitude evaluation, the maximum allocated users were 45 (Figure 4a). Moreover, results show how the network usage was reasonably stable and 80 m flight-altitude outcast other altitudes, showing that our tool enabled a stable backhaul connection.

### 5.2. Scenario II: LTE at 3.5 GHz with Carrier Aggregation

**Figure 5:**
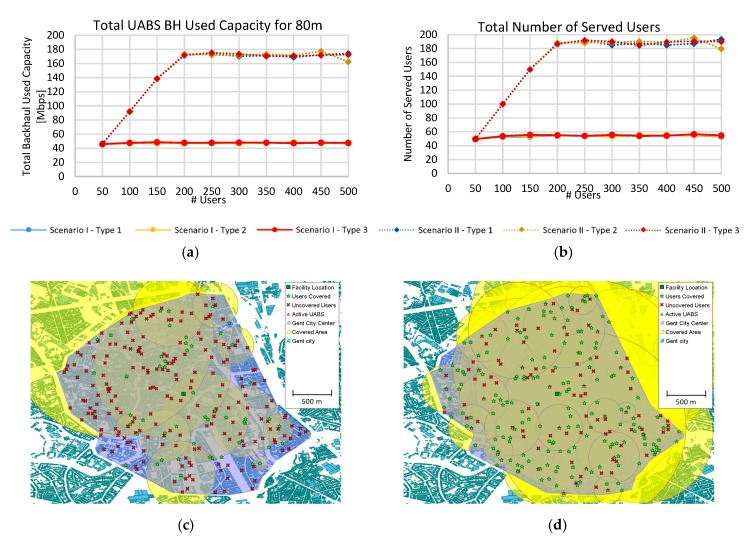
Evaluation results for Scenario II. (**a**) Used capacity of the backhaul for 80 m flight height. (**b**) Provisioned users. (**c**) Network coverage for 260 users at 80 m flight height Type 2 drone in Scenario I. (**d**) Network coverage for 260 users at 80 m flight height Type 2 drone in Scenario II (carrier aggregation). Facility location (dark green square); users covered (green star); uncovered Uuers (red cross); active UABS (orange triangle); covered area (yellow circle).

The previous scenario shows that the network was saturated due to the full usage of resource blocks. To overcome this situation, carrier aggregation (CA) the land-based system severely where five contiguous component carriers of 20 MHz each were aggregated for a total of 100 MHz and 500 RB. Figure 5a compares the backhaul network capacity without (Scenario I) and with CA (Scenario II). The first scenario only used 100 RB while the second 500 RB. All the network resources were allocated at about 190 users assigning nearly 175 Mbps, and the capacity usage was independent of the type of drones. Moreover, when the available RB five-folds, the used capacity only incremented 3.7 times (47.6 Mbps to 173.1 Mbps). This is because our algorithm organized the best suitable UABS with higher SNR and served them first. Hence, after the first 100 RBs were allocated, the best UABSs were served and now UABSs with lower SNR and worst MCS were served, decreasing the overall efficiency of the backhaul link. Similarly, the number of covered users incremented 3.5 times (from 54 users to 189 users), but after 200 requested users the number of served ones was constant due to RB saturation in the backhaul as shown in Figure 5b. Likewise, the number of UAV used increased only 1.4 times (from 18.5 T2 drones without CA to 26.5 T2 drones with CA) leading to an optimization of the backhaul and an improvement of the mean served users by each UABS when using CA. 

In Figure 5c,d, we presented the results of the coverage in the real scenario in the city of Ghent. Green stars are the served users, while the red crosses are the uncovered users. Figure 5c shows that even when a user could have a viable connection in path loss terms, it was not served due to backhaul capacity restrictions. When CA was used in Figure 5d, the number of users served was 354.3% higher and the average distance of UABS locations increased leading to more distant UABSs from the central facility as seen by the orange triangles. 

### 5.3. Scenario III: LTE with a Millimeter-Wave Backhaul Link

**Figure 6:**
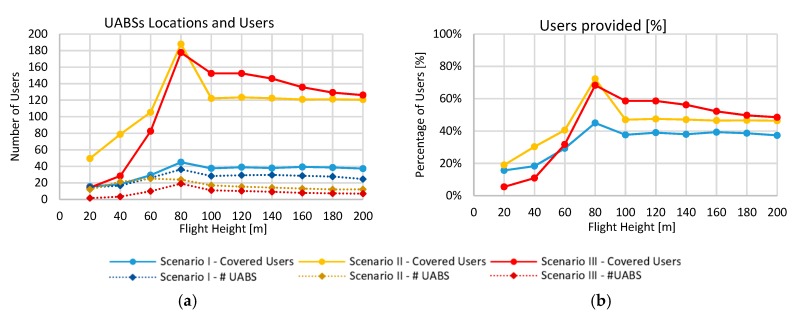
Comparison of network performance in different flight heights. (**a**) Number of users and number of UABS. (**b**) Percentage of served users.

Although the usage of carrier aggregation as described in the previous scenario, not all the users could be allocated due to the network restrictions. The proposed solution was to use the 60 GHz band to support uncovered users in a disaster scenario. In Figure 6, a comparison of the three backhaul link scenarios was presented. Here, it can be seen that the number of locations and served users for the CA and millimeter-wave was comparable. For lower altitudes, carrier aggregation could serve slightly more users, and for higher elevations, the millimeter backhaul link could serve more. At the optimal altitude of 80 m, CA performed better, serving 188 users compared to the 177 using 60 GHz. It is known that the 60 GHz band has higher path losses, especially when NLoS links are presented, mainly due to building losses. In some simulations runs (up to 16 runs 24.5%), no UABSs could be connected to the facility, particularly in elevations under 40 m due to high path losses through buildings. This can be easily overcome by incrementing the flight altitude of the drone, as shown in Figure 6b. However, despite that 80 m height was the best altitude, only 68.3% of the users were covered. This is because only UABSs locations in a radius of 550 m close to the facility were served. According to the link budget calculations, if a drone is in an NLoS using the 60 GHz band, the maximum distance will be 390 m. However, the UABSs between 390 m and 550 m, are connected through LoS where the angle is high enough to avoid buildings.

**Figure 7:**
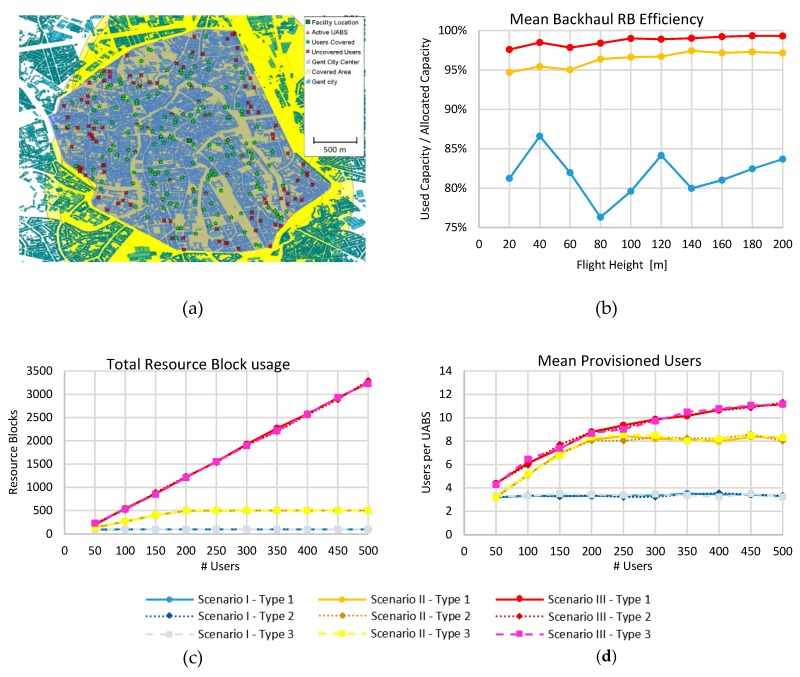
Performance of 60 GHz band and comparison with 3.5 GHz scenarios. (**a**) Coverage using the 60 GHz band. (**b**) Backhaul resource block (RB) efficiency. (**c**) Resource block used. (**d**) Provisioned users.

In Figure 7a, the coverage simulation results in the city of Ghent for the 60 GHz band are presented. In this case, all the users in a distant radius of 750 m from the facility were served due to the coverage extension obtained by the UABSs. Some users far away from the facility were connected to UABSs that had LoS link to the facility. In comparison with CA, 60 GHz connections outperformed the served users in altitudes higher than 100 m, where NLoS connections were minimal. In Figure 7b, the efficiency of the backhaul link as a function of the flight height is shown. It is shown that the performance of 60 GHz (98.7%) was better than CA (96.4%). This is because the MCSs for the 3.5 GHz band could allocate bigger data packets in the RB, leading to a difficult resource allocation that was simplified in the 60 GHz allocation process. This goes with the cost of using up to four times more RB than in the 3.5 GHz band. Furthermore, the 60 GHz backhaul link had only three schemes with higher SNR values, compared with the seven in the 3.5 GHz band. It used better connections that led to overall better results in resource allocation.

Next, we evaluated how the demand of users affected the network (Figure 7c,d). The results were proportional to the number of demanding users because the number of RBs for the 60 GHz network was much higher (45000 RBs) compared to the maximum users in our simulation and backhaul saturation was not achieved. For 500 demanding users, only 3290 RBs were needed, which was 7.3% of the total RBs at 60 GHz. Figure 7c presents the usage of RB for the three scenarios. It shows that for the 3.5 GHz band, the number of the RBs was fully used, but for the 60 GHz there was space for more.

On the other hand, the backhaul RB efficiency was stable at around 98.1% ± 0.8%. In Figure 7d, the number of users served by each UABS is presented. It is shown that CA and 60 GHz performed quite similar for less than 200 users. From 200 users on, the CA was saturated when having eight users per UABS, but for 60 GHz, it increased more gently. However, using 60 GHz, the percentage of covered users was smaller than 72% due to the propagation restrictions described previously. The capacity results show that using the simultaneous resource allocation could lead to a feasible solution, serving up to 54 (21.6%), 188 (72.4%) and 178 (68.5%) users for a 3.5 GHz, 3.5 GHz with CA and 60 GHz network configurations, respectively.

Finally, we presented in Table 4 a resume of the principal results of the network performance for the three scenarios with three types of drones for 260 users and the optimal flight height. Here it can be seen how Scenario II could support more users (188 users) and slightly outperformed the scenario of the 60 GHz band (178 users). Contrarily, in scenario III, each UABS could support more users (10 users in III and eight in II). As expected, the usage of better drones (type 2 and type 3) reduced the number of drones required in all three scenarios by a factor of four, being the hybrid-type was slightly (11%) better than type 2. The power consumption was also stable among the kinds of drones and quite different among the scenarios, concluding that power consumption was mainly related to the technology and network requirements rather than drone flight usage. It can be seen that for the scenarios I and II, the power consumption per UABS was nearly 34 and 36 watts respectively, meanwhile, for scenario III, it was just 25 watts.

Moreover, the total capacity was consequent with the number of users served and scenario II was the one that outperformed with nearly 173.1 Mbps over 163.8 Mbps and 47.6 Mbps for scenarios III and I respectively. Similarly, to the more users per UABS, scenario III could support more backhaul bitrate per UABS with 8.8 Mbps. Next, due to the differences in the MCS for both frequencies, the 60 GHz band had less spectrum efficiency. Hence, it consumed much more resource blocks compared with the 3.5 GHz band. In can be seen that scenario I and II were saturated in 100 and 500 RB, but for scenario III, it consumed almost 1620 RB still far from its 45000 RB limit. In this case, the scenario I had more RB capacity with 476.5 kbps/RB followed by scenario II and III with 346.2 kbps/RB and 101.2 kbps/RB respectively. Lastly, the RB efficiency increments as the number of RB per UABS were used. This is due to the optimization algorithm that organized the UABS capacity in a better way leading to an average of 87.1%, 96.2% and 98.5% of efficiency for scenarios I, II and III respectively.

## 6. Conclusions and Future Work

Despite that land-based networks are quite reliable, in case of an emergency, they could suffer from saturation or total failure. Using fast deployable systems mounted on UAVs are a promising solution, but they arise challenges as optimal drone selection and placement, radio network section including the access and backhaul. This paper presented a network architecture based on UAVs to provide wireless connectivity for a disaster scenario and the performance evaluation of the capacity for the proposed architecture. To this end, we developed a novel simulation tool and applied it on a realistic scenario in the city center of Gent in Belgium, accounting for capacity and power restrictions from both the access and backhaul networks. The study focused on the usage of unoccupied frequency bands such as 3.5 GHz and 60 GHz and compared the performance of variables such as the number of demanding users and UABSs’ flight altitude.

The study also introduced the comparison of traditional off-the-shelf drones with hybrid ones and concluded that the last one outperformed in flight time and power usage. Further analysis in the weights of the backhaul equipment needs to be done in the future. Our results showed that for the proposed architecture, 80 m was the optimal flight altitude. The capacity results showed that by using the simultaneous access and backhaul resource allocation a real solution could be achieved, serving up to 17.3%, 72.4% and 68.1% of the users for a 3.5 GHz link, 3.5 GHz with carrier aggregation and 60 GHz network configuration, respectively. For the 60 GHz link, the main limitation of users served was due to the distance as a result of path loss building penetration reducing the covered area down to a radius of 750 m. Contrarily, the 3.5 GHz solutions were mainly limited by the full usage of resource blocks.

Future work will include the usage of a multi-frequency backhaul network using 3.5 GHz and 60 GHz bands choosing which link is optimal to serve UABSs with different constraints. Moreover, future work may involve the usage of Multiple-Imput Multiple-Output (MIMO) antennas, multi-channeling to reduce the noise floor and beamforming analysis. Investigation when part of the terrestrial network is down, might also be included, where inter-cell interference is evaluated in conjunction with in-band an out-of-band radio resource allocations. Different architectures that can be included in future studies are the usage of relay network, including a low-altitude aerial platform (LAP) or satellite as suggested in the literature with all its implications like internode interference and multi-antenna devices. 

## Figures and Tables

**Table 1:** Scenario simulation parameters.

Parameter	Value
Area size	6.85 km^2^ (Ghent, suburban)
Number of users/devices	260 users
User distribution	Uniform
Traffic demand	1 Mbps data/64 kbps voice
Facility size	1500 drones
Intervention time	1 h

**Table 2:** Link budget parameters for the simulation.

Parameter	Sub 6 GHz Backhaul	mmWave Backhaul
Frequency	3.5 GHz	61.5 GHz
Bandwidth	20 MHz	9 GHz
Number of resource blocks	100	45,000
Number of used subcarriers	1200	540,000
Total number of subcarriers	2048	1,048,576
Max transmission power UABS	43 dBm	10 dBm
Max transmission power CN	43 dBm	10 dBm
Antenna gain UABS	5 dBi	36 dBi (2.5°)
Antenna gain CN	5 dBi	36 dBi (2.5°)
Fade margin	10 dB	5 dB
Interference margin	2 dB	2 dB
Receiver signal-to-noise ratio (SNR) for the modulation and coding scheme (MCS)	1/3 QPSK = –1.5 dB 1/2 QPSK = 3 dB 2/3 QPSK = 10.5 dB 1/2 16-QAM = 14 dB 2/3 16-QAM = 19 dB 1/2 64-QAM = 23 dB 2/3 64-QAM = 29.4 dB	½ BPSK = 7.39 dB ½ QPSK = 15.4 dB ½ 16 QAM = 17.5 dB
Noise figure in UABS	5 dB	5 dB
Shadowing margin	8.2 dB	8.2 dB
MIMO gain	0 dB	0 dB
CN antenna height	25–60 m	
User height	1.5 m	

**Table 3:** Drone specification for simulation.

Drone	Type 1	Type 2	Type 3 Hybrid
Average UAV speed (m/s)	15	12	15
UAV battery capacity (Ah)	2	17.33	100
UAV battery voltage (V)	14.3	22.2	48.0
UAV average usage (A)	5	13	25
UAV average usage (W)	71.3	288.6	1200
Average Max Fight Time (s)	900	2400	7200
Fly height	Uniformly distributed between 20 m and 200 m		

**Table 4:** Resume of the critical parameters for 260 users at 80 m drone altitude.

	Scenario I			Scenario II			Scenario III		
	Type 1	Type 2	Type 3	Type 1	Type 2	Type 3	Type 1	Type 2	Type 3
**USERS**									
Users served (users)	54	54.2	54.1	188.2	187.9	190.8	177.6	178.1	177.6
Users served (%)	21.6	21.7	21.7	72.4	72.3	73.4	68.3	68.5	68.3
Users per UABS	3.36	3.2	3.4	8.1	7.9	8.1	9.4	10.1	9.6
**UABSs**									
# UABSs locations	16.4	17.0	16.2	23.4	23.9	23.6	19.2	18.6	18.8
# Used UABS	75.2	18.5	16.2	110.1	26.5	23.6	76.7	18.6	18.8
Mean power usage (w)	34.4	33.7	35.3	36.9	36.2	36.9	24.9	24.9	24.9
**CAPACITY**									
Total BH capacity (Mbps)	47.4	47.6	47.9	173.1	171.4	174.7	163.8	164.1	163.5
BH capacity per UABS (Mbps)	2.9	2.8	2.9	7.4	7.2	7.4	8.7	8.8	8.81
Total RB usage (RB)	100	99.9	100	500	499.8	499.9	1617.8	1619.1	1620.8
RB usage per UABS (RB)	6.2	5.9	6.2	21.5	21.0	21.3	85.4	87.1	87.36
RB capacity (kbps/RB)	474.0	476.5	479.0	346.2	342.9	349.5	101.2	101.3	100.8
BH RB efficiency (%)	87.0	87.2	87.1	96.2	96.4	96.1	98.4	98.7	98.5
